# Promoting faculty professionalism: a case-based approach

**DOI:** 10.1007/s40037-015-0204-x

**Published:** 2015-07-17

**Authors:** Patricia M. Dieter, Nicholas M. Hudak, Peggy R. Robinson

**Affiliations:** Duke University School of Medicine, DUMC 104780, 27710 Durham, NC USA

**Keywords:** Faculty, Professionalism, Boundaries

## Abstract

**Introduction:**

Professionalism is a key attribute for health professionals. Yet, it is unknown how much faculty development is directed toward skills and behaviours of faculty professionalism. Faculty professionalism includes boundaries in teacher-student relationships, self-reflection, assuring one’s own fitness for duty, and maintaining confidentiality when appropriate.

**Methods:**

For five years, we have incorporated faculty professionalism as a routine agenda item for the monthly Physician Assistant Programme faculty meetings, allowing faculty members to introduce issues they are comfortable sharing or have questions about. We also have case discussions of faculty professionalism within faculty meetings every three months.

**Results:**

Faculty professionalism is important in the daily work lives of faculty members and including this as part of routine agendas verifies its importance. A faculty survey showed that a majority look forward to the quarterly faculty professionalism case discussions. These have included attempted influence in the admissions process, student/faculty social boundaries, civic professionalism, students requesting medical advice, and self-disclosure.

**Conclusion:**

A preventive approach works better than a reactionary approach to faculty missteps in professionalism. Routine discussion of faculty professionalism normalizes the topic and is helpful to both new and experienced faculty members. We recommend incorporation of faculty professionalism as a regular agenda item in faculty meetings.

## Introduction

Professionalism is a core competency for providers and taken seriously by faculty in developing student behaviours [1, 2]. Accreditors in the United States, including the Licensing Commission on Medical Education (LCME), the Accreditation Commission for Education in Nursing (ACEN), and the Accreditation Review Commission on Education for the Physician Assistant (ARC-PA), highlight professionalism within their accreditation standards. Similar development for faculty may include offerings dealing with professionalism, but these classes are usually aimed at shaping professionalism in others [3–5].There is an institution-wide requirement at Duke for departmental faculty to periodically discuss issues of professionalism. The specifics of how and when to address this requirement are left to the discretion of each department. We found that with divergent clinical, teaching and administrative responsibilities, our faculty often fulfilled the requirement on a more individual rather than a collective basis. As we began to incorporate issues of professionalism into our students’ professional curriculum we realized that faculty rarely had dedicated discussions on this issue. We had, in effect, a reactive approach; issues of professionalism among the faculty were rarely discussed as a group unless a problem arose.

## Methods

Our educational programme chose to fulfil Duke’s requirement for periodic discussion of issues of faculty professionalism through a unique approach. Routine faculty discussions of various professionalism topics occur at monthly general faculty meetings that all faculty are expected to attend. At each meeting, we allot time for individuals to present professionalism issues to colleagues for the purpose of raising awareness of issues and to develop an approach to resolve dilemmas.

Also, on a quarterly basis, there is additional time for deliberate discussion of a case example involving a professionalism issue from the faculty perspective. Approximately 15 min of the meeting are allocated in the agenda for this purpose. Each discussion is facilitated by a different member of the faculty so as to directly involve many faculty members in the process and to encourage variation in the professionalism issues to be presented. To ensure the collaborative nature of these activities, faculty members volunteer to present a professionalism issue of their own choosing. Self-selection of a topic provides every faculty member an opportunity to present an issue that may be either of unique concern or more applicable to the entire faculty.

**Fig. 1:**
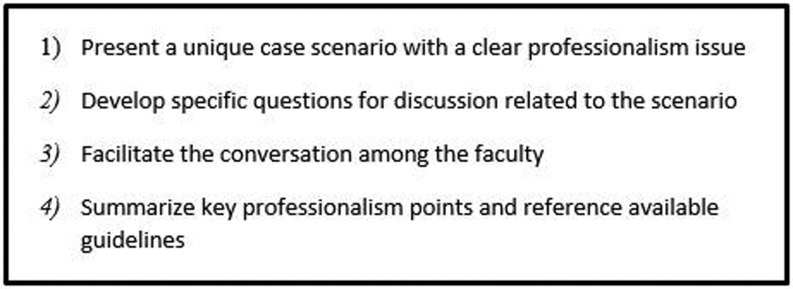
Facilitator guidelines for professionalism discussion

The responsibilities of the facilitator involve the following: (1) presenting a unique case scenario with a clear professionalism issue, (2) developing specific questions for discussion, (3) facilitating the conversation among the faculty, (4) summarizing key professionalism points and referencing available guidelines (Fig. 1). Customarily, facilitators have several months to prepare in advance of the discussion, as well as having the opportunity to consult with senior faculty members for guidance. During each discussion, all faculty members are encouraged to engage in conversation that aims to describe professional approaches and responses to the presented scenario. Following each discussion, the facilitator may provide additional resources related to the professionalism topic and outcomes of the group discussion. For some cases, facilitators may reference recommendations from the literature or guidelines from professional organizations. Faculty discussion of professionalism topics is recorded in the meeting minutes and a list of previously presented topics is kept by programme leadership for future reference.

A majority of faculty members have facilitated one case discussion and the longevity of this approach now affords faculty additional opportunities to present new professionalism topics. Over the past five years of implementation, there have been no significant changes to the approach. A survey was recently conducted to evaluate faculty impressions and acceptance of the approach.

## Results

**Fig. 2:**
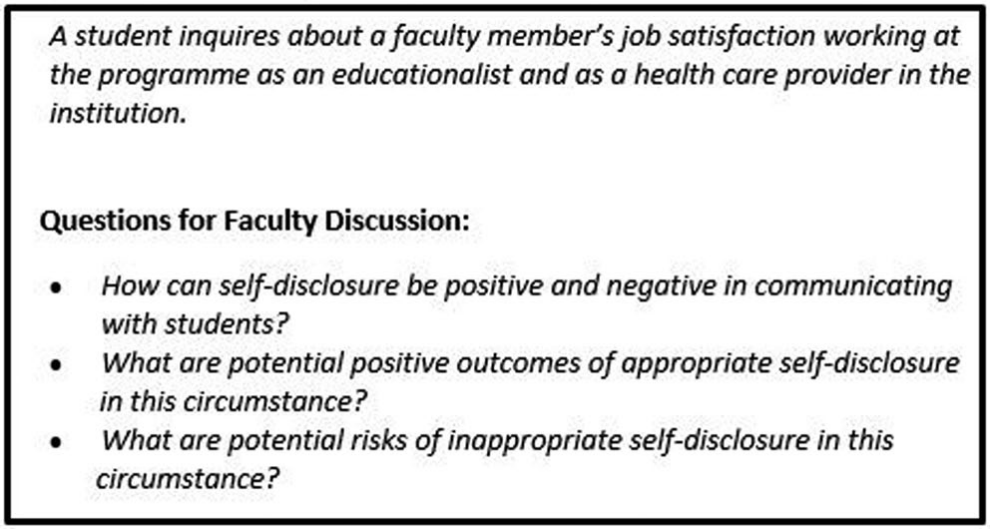
Case example

Our approach has effectively addressed the need to routinely discuss faculty professionalism; we are now proactive in our discussions. Essential to the success of this change is its collaborative nature. There has been uniform buy-in. The case approach allows for discussion of broader professionalism issues as well as those of particular concern or relevance based on an occurrence within the academic setting (Case Example, Fig. 2).

We administered a brief survey to faculty via Qualtrics, three years into our new approach. The survey and intended use of data was submitted to the Duke Institutional Review Board and received exempt status. Fourteen of 17 faculty members (82.4 %) responded to the survey. The results validate the initiative and show that faculty find the professionalism discussions useful (Table 1).

**Table 1:** Faculty survey responses: 14/17

	Strongly disagree/disagree (%)	Agree/Strongly agree (%)
The faculty professionalism cases have provided new information	25	75
The faculty professionalism cases are relevant to my work as a faculty member	0	100
The faculty professionalism cases help me evaluate my professionalism on an ongoing basis	25	75

The range of topics covered helps to clearly delineate the breadth of issues that fall within the professionalism realm.

Many times the topics are insightful and right on target with current issues.

I find the cases pertinent and thought provoking.

I always appreciate the opportunity to reflect on issues that may change over time and to hear the views and expertise of other faculty members.

Since the implementation of our initiative we have had lively discussions on a variety of topics including boundary issues, new certification policies and in the wake of new state legislation the role of civic professionalism among health professionals. This allows a forum for every faculty member to present a particular topic of interest. It is important to note that our quarterly structured approach does not preclude discussion of any pertinent issues at monthly general faculty meetings.

## Discussion

Our novel initiative can be easily duplicated at other institutions where no forum for discussions of faculty professionalism exists. Carving out dedicated time for discussion of a wide range of professionalism issues is critical for many reasons. It creates an atmosphere of the expectation of professionalism for all faculty members; this is important for both new and experienced faculty members. It keeps professionalism in the forefront and provides us as faculty the opportunity to model it for our students. Thus, it is no longer a set of vague ideals we encourage them to adopt but a culture of interacting with each other and with them that is visible.

Dedicated time prevents neglecting or postponing these discussions. There are occasions when more questions than answers arise or a topic generates the desire for more extended conversation. These issues can then be carried over into faculty retreats or explored by faculty subcommittees and reintroduced at subsequent meetings.

## Conclusion

Our faculty and departmental leadership have been very pleased with the outcome of our unique case-based approach to keeping issues of faculty professionalism in routine discussion. Existing literature is thin on approaches to faculty professionalism. Medical educators may benefit from future articles describing other approaches to promoting faculty professionalism as well as research reporting faculty satisfaction with professional development activities and any related impact on professionalism as a competency.

### Ethical approval

Ethical approval for the survey conducted as part of this article was granted by the Duke University Medical Center Institutional Review Board (Exempt Status).

### Conflict of interest

The authors report no declarations of interest.
